# ASCO 2017: highlights in breast cancer

**DOI:** 10.1007/s12254-017-0368-7

**Published:** 2017-11-13

**Authors:** Rupert Bartsch, Elisabeth Bergen

**Affiliations:** 1 Department of Medicine 1, Clinical Division of Oncology, Medical University of Vienna, Waehringer Guertel 18–20, 1090 Vienna, Austria; 2 Comprehensive Cancer Center Vienna, Medical University of Vienna, Vienna, Austria

**Keywords:** ASCO Annual Meeting 2017, Breast cancer, Highlights, Review

## Abstract

At the 2017 ASCO Annual Meeting, several pertinent studies in the field of breast cancer were presented and some are deemed as being potentially practice changing. BrighTNess was the first phase III study to investigate the addition of carboplatin to standard neoadjuvant chemotherapy in triple-negative breast cancer; while toxicity was increased in the experimental group, a significantly higher pathologic complete remission (pCR) rate was observed as well suggesting that adding carboplatin to neoadjuvant anthracycline, cyclophosphamide and taxane-containing regimens is efficacious in otherwise healthy patients. In metastatic breast cancer patients harbouring *BRCA* germ-line mutations, the PARP(poly [ADP-ribose] polymerase)-inhibitor olaparib was superior to conventional chemotherapy defining a potential novel treatment standard in this high-risk population. In the adjuvant setting, the APHINITY trial compared dual HER2-directed antibody therapy with trastuzumab plus pertuzumab to trastuzumab alone. A small benefit in favour of the combination was observed which was more pronounced in node-positive subjects. In hormone-receptor positive metastatic disease, several studies evaluating the role of CDK4/6 (cyclin-dependendent kinases 4 and 6) inhibitors were presented with data again indicating that adding CDK4/6 inhibitors to endocrine therapy results in a clinically relevant prolongation of progression-free survival.

## Neoadjuvant chemotherapy

In the neoadjuvant treatment of triple-negative breast cancer (TNBC), the exact role of carboplatin is still debated; two important trials in this field were presented at this year’s ASCO Annual Meeting. The phase III GeparOcto study [1] randomized 961 patients (43% TNBC; 46% N+) to a GeparSixto [2] style regimen of weekly non-pegylated liposomal doxorubicin, paclitaxel and carboplatin (P[Cb]) or an intensified dose-dense (idd) regimen of sequential epirubicin (150 mg/m^2^), paclitaxel (225 mg/m^2^) and cyclophosphamide (2000 mg/m^2^) each given for three cycles (EPC); trastuzumab plus pertuzumab were added in the HER2-positive population. Pathologic complete remission rates (pCR) were high and similar in between both groups (PM[Cb] 47.6%; EPC 48.3%) in the entire population as well as in the subset of patients with TNBC (51.7% *vs*. 48.5%). On the other hand, a high rate of treatment discontinuations was seen in both arms with numbers favouring EPC (PM[Cb] 33.8%; EPC 16.4%); in addition, two toxicity-related deaths were recorded.

When considering these results, it needs to be mentioned that GeparOcto, as GeparSixto, added carboplatin to a non-standard cyclophosphamide-free neoadjuvant regimen. Due to this fact as well as due to the non-standard idd comparator arm, this large study is not able to provide conclusive evidence on the potential role of neoadjuvant carboplatin.

The BrighTNess study, on the other hand, was the first placebo-controlled phase III trial to evaluate the addition of carboplatin (with or without veliparib) to a standard neoadjuvant chemotherapy backbone consisting of paclitaxel weekly × 12 followed by four cycles of AC once every two or three weeks [3]. Overall, 634 TNBC patients were randomized 2:1:1 to carboplatin AUC6 plus veliparib plus standard chemotherapy (arm A), carboplatin plus standard chemotherapy (arm B), or standard chemotherapy alone (arm C). Only patients with large (T2 to T4) tumours and/or node-positive disease were included.

While toxicity was significantly increased by the addition of carboplatin (Grade 3–4 AEs arm A 86%; arm B 85%; arm C 45%), pCR rates were significantly higher as well (pCR rate arm A 53.2%; arm B 57.5%, arm C 31%; arm A *vs.* C *p* < 0.001; *post hoc* analysis arm B *vs.* C *p* < 0.001). Therefore, in a general population of TNBC patients not selected for *BRCA* mutations, the PARP inhibitor veliparib provided no additional benefit over chemotherapy; addition of carboplatin, on the other hand, significantly increased pCR rates and may now be considered as a reasonable option in healthy TNBC patients scheduled to receive neoadjuvant chemotherapy consisting of anthracyclines, taxanes and cyclophosphamide.

A retrospective analysis of six neoadjuvant trials conducted by the German Breast Group evaluated if the time from biopsy to chemotherapy initiation (TBC) or from chemotherapy to surgery (TCS) was associated with long-term outcome [4]; a cut-off of < 4 weeks or ≥4 weeks was chosen.

In total, 9127 data sets were analysed, approximately half of these patients were node-positive and one quarter was diagnosed with TNBC. TBC was not a significant predictor of pCR, disease-free survival (DFS) or overall survival (OS) while a trend towards superior DFS in the TCS < 4 weeks group was observed both in the overall population (HR 1.11; 95% CI 0.99–1.24; *p* = 0.08) as well as in patients without pCR (HR 1.12; 95%CI 0.99–1.26; *p* = 0.08) suggesting that a shorter TCS may be beneficial.

## Immunotherapy

In contrast to many other malignant diseases, the potential role of immunotherapy in breast cancer is still ill defined.

The prospective phase II study KEYNOTE-086 evaluated the role of pembrolizumab in metastatic TNBC [5]. Two separate patient cohorts—pretreated for metastatic disease (cohort A) and previously untreated (cohort B)—received the PD-1 antibody pembrolizumab at a dose of 200 mg once every three weeks. Treatment was well tolerated but activity of single-agent immunotherapy was disappointing with only 4.6% of patients in cohort A responding to therapy; median progression-free survival (PFS) was two months (95% CI 1.9–2.0) and no difference in terms of response rate between patients with PD-L1 positive and PD-L1 negative tumours was observed. In a separate analysis, preliminary outcome data of 52 first-line patients in cohort B was reported; in treatment-naïve patients, the response rate was higher at 23% [6].

These data again suggest that single-agent immunotherapy is apparently not efficacious in the majority of metastatic breast cancer cases. In contrast, a combination of immune checkpoint modulators with chemotherapy apparently holds great promise as shown in KEYNOTE-173 [7]. In this phase Ib trial, pembrolizumab was added to conventional neoadjuvant chemotherapy consisting of weekly nab-paclitaxel × 12 followed by four cycles of AC (with or without carboplatin AUC6 once every three weeks added to nab-paclitaxel). Ten patients were treated in each cohort; corresponding pCR rates in breast and axilla were 50% in the non-carboplatin cohort (90% CI 22–78%) and 90% in the carboplatin cohort (90% CI 61–100%); as expected, toxicity was higher in the carboplatin group. Of note, no dose-limiting toxicity linked to pembrolizumab was observed. Naturally, no final conclusions can be drawn from these results; still data suggest that the combination of immunotherapy with chemotherapy should be further investigated in TNBC.

## Olaparib in metastatic TNBC harbouring *BRCA* mutations

Despite the great interest in the field of immunotherapy, the OlympiAD trial was perhaps the most relevant of all breast cancer studies presented at the 2017 ASCO Annual Meeting [8]. In this open-label phase III trial, 302 HER2-negative metastatic breast cancer patients harbouring germ-line *BRCA* mutations were randomly assigned to the PARP-inhibitor olaparib or treatment by physician’s choice (TPC) consisting of either capecitabine, vinorelbine or eribulin.

Median patient age was low at 44 years, and 50% had TNBC; 71% were pretreated with chemotherapy for metastatic disease and more than one quarter had already received platinum salts. PFS was significantly longer in the olaparib group (7 *vs*. 4.2 months; HR 0.85; 95% CI 0.43–0.80; *p* = 0.0009); furthermore, time to second progression (PFS2) was significantly longer in patients receiving olaparib as well (HR 0.57; 95% CI 0.40–0.83). Of note, the superiority of olaparib was even more pronounced in patients with TNBC and those without prior platinum exposure and response rates—potentially relevant in high-risk breast cancer subtypes—were higher in the olaparib group as well. Furthermore, less ≥3 AEs were observed (36.6% olaparib *vs. *50.5% TPC) and superior quality-of-life was improved as well. Based upon these facts, olaparib is regarded as clinically relevant addition the therapeutic armamentarium in patients with metastatic breast cancer harbouring germ-lin*e BRCA* mutations.

## Adjuvant treatment of HER2-positive breast cancer

Currently, dual HER2-inhibition with trastuzumab plus pertuzumab and chemotherapy is regarded as the standard-of-care in the neoadjuvant treatment of HER2-positive breast cancer while in the adjuvant setting, trastuzumab for a total duration of one year is recommended.

The phase III APHINITY trial investigated the role of pertuzumab when added to trastuzumab after surgery and chemotherapy in the adjuvant setting [9]. Overall, 4805 patients were randomized to pertuzumab or placebo, two thirds of whom were node-positive (63%). At three years, 94.1% of patients were free of invasive disease in the pertuzumab group as compared to 93.2% in the placebo group (HR 0.81; 95% CI 0.66–1; *p* = 0.045). This result was driven by node-positive patients (92% *vs*. 90.2%; HR 0.77; 95% CI 0.62–0.96, *p* = 0.019) while in the node-negative subset no difference was seen. Therefore, adjuvant dual HER2-inhibiton may be considered in HER2-positive breast cancer patients with upfront surgery and positive nodes.

The optimal duration of postoperative trastuzumab is a matter of ongoing research. Currently, one year of therapy remains the standard-of-care but several groups have investigated shorter course adjuvant regimens. At the 2017 ASCO Annual Meeting, results of the Italian ShortHER trial were presented [10]. This phase III trial randomized patients to standard treatment consisting of AC or EC × 4 followed by four cycles of docetaxel and 18 cycles of trastuzumab once every three weeks or three cycles of docetaxel in combination with nine administrations of weekly trastuzumab followed by three cycles of FEC. Therefore, chemotherapy intensity was lower as well. In total, 1254 (46% node-positive) of a planned number of 2500 patients could be accrued, thereby limiting statistical power. Non-inferiority was defined as primary study-endpoint with a HR of <1.29 as definition of non-inferiority.

The five-year, disease-free survival (DFS) was 87.5% in the standard group and 85.4% in the short-course group (HR 1.15) but confidence intervals crossed the non-inferiority boundary (90% CI 0.91–1.46); therefore, non-inferiority could not be formally established. Overall survival (OS) was identical at 95 and 95.1%, respectively. In patients with stage III disease and/or >3 positive nodes, however, a significant benefit in favour of long-term therapy was observed. As expected, the number of cardiac events was lower in the short-course group (90 *vs.* 32). Therefore, one year of trastuzumab remains the standard-of-care but shorter course therapy may be an option in selected patients with low clinical stage and significant cardiac risk.

## HER2-positive MBC

No practice-changing data were presented at this year’s ASCO Annual Meeting in the field of HER2-positive metastatic breast cancer with chemotherapy plus dual HER2-inhibition consisting of trastuzumab plus pertuzumab remaining the standard-of-care in the first-line setting and T‑DM1 as standard second-line therapy. Of note, T‑DM1 was never formally tested in patients receiving trastuzumab plus pertuzumab as first-line therapy.

In a post hoc analysis, Urruticoechea et al. investigated the activity of T‑DM1 in patients pretreated with pertuzumab pus trasutzmab within the CLEOPATRA or PHEREXA studies [11]. Median duration of T‑DM1 therapy was 7.1 (range 0–44) and 4.2 (range 0–22) months respectively, and OS was numerically longer in patients receiving T‑DM1 after progression on trastuzumab plus pertuzumab (CELOPATRA: 39.6 *vs.* 46.2 months; HR 0.93; 95% CI 0.58–1.49; *p* = 0.7538; PHEREXA: 23.7 *vs.* 40.1 months; HR 0.45; 95% CI 0.26–0.81; *p* = 0.0061). While OS data are potentially biased by the fact that patients living longer after progression on study treatment had a higher probability of receiving T‑DM1 during their later course of disease, these data still indicate a clinically relevant activity of T‑DM1 in patients progressing on prior dual antibody therapy.

Activation of the PI3K/mTOR/akt pathway was identified as a potential mechanism of resistance to HER2-directed therapy. A phase I trial therefore investigated the combination of the alpha-specific PI3K inhibitor BYL719 (alpelisib) with T‑DM1 3.6 mg/m^2^ once every three weeks [12]. Seventeen patients (median number of prior treatment lines 4.5) were included. At the initial dose of 300 mg BYL719 daily, 2/5 patients experienced a dose-limiting toxicity (DLT) while no DLTs were observed at level −1 (BYL719 250 mg); main toxicities associated with PI3K inhibition consisted of hyperglycaemia, fatigue, nausea and rash. In this heavily pretreated population, a median PFS of six months was reported (95% CI 2.9–10.6); of note, in six patients with prior progression on single-agent T‑DM1, PFS was 10.6 months (95% CI 1.6–12.6). This suggests that reversal of resistance may indeed be possible with the addition of BY719 to HER2-directed therapy and further investigation of this strategy appears warranted.

## Adjuvant endocrine therapy

Based upon preclinical data, intermittent hormone withdrawal with an aromatase-inhibitor may be superior to standard (continuous) treatment. Therefore, the phase III SOLE trial randomized 4884 patients after five years of adjuvant endocrine therapy to another five years of continuous letrozole or an intermittent regimen of nine months on–three months off for four years followed by 12 months of continuous treatment in the last year [13].

At 60 months median follow-up, the five-year DFS was 87.5% in the standard arm and 85.8% in the experimental arm (HR 1.08; 95% CI 0.93–1.26; *p* = 0.31) with similar results observed for breast cancer-free interval (HR 0.98), distant recurrence-free interval (HR 0.88) and OS (HR 0.85). Of note, a trend favouring continuous therapy in patients with prior tamoxifen therapy was observed while in patients with prior adjuvant AI-only therapy intermittent treatment seemed beneficial; this difference, however, did not reach statistical significance. Quality-of-life was superior in the intermittent therapy group suggesting that temporary therapy interruptions may be acceptable in selected patients.

## Hormone-receptor positive metastatic breast cancer

Finn et al. presented updated results of the phase II PALOMA-1 study comparing a combination of letrozole plus the CDK4/6 inhibitor palbociclib to letrozole alone as first-line therapy [14, 15]. As already published, combination therapy resulted in a clinically relevant and statistically significant prolongation of PFS from 10.2 to 20.2 months (HR 0.488; 95% CI 0.319–0.748; *p* = 0.0004); OS, however, was similar between the two groups (37.5 *vs.* 34.5 months; HR 0.897; 95% CI 0.623–1.294; *p* = 0.281). Due to the small size of this phase II study, however, no conclusion regarding a potential survival benefit can be drawn. Importantly, time to chemotherapy was numerically longer in the combination group hinting at the clinical relevance of longer PFS in a population with metastatic luminal breast cancer (17.7 *vs*. 26.7 months).

In addition to PALOMA-1, updated results from the MonaLEEsa-2 study were presented as well. In this placebo-controlled phase III trial conducted in the first-line setting, 668 patients were randomly assigned to letrozole with or without ribociclib, another CDK-4/6 inhibitor [16]. At a median follow-up of 26.4 months, median PFS was 25.3 months in the combination group as compared to 16 months on letrozole plus placebo (HR 0.566; 95% CI 0.457–0.704). While OS data remain immature, a trend towards prolonged OS was observed in favour of the experimental arm as well (HR 0.746; 95% CI 0.517–1.078; *p* = 0.059). As expected, the main toxicity in patients receiving the CDK4/6 inhibitor was neutropenia.

Finally, in the field of CDK4/6 inhibitors, data from the MONARCH-2 trial were presented [17]. Overall, 669 patients who had relapsed on adjuvant endocrine therapy or within twelve months from the end of adjuvant endocrine therapy or progressed on first line endocrine therapy were accrued to this phase III study and randomly assigned (2:1) to fulvestrant plus/minus the CDK4/6 inhibitor abemaciclib. Due to a high diarrhoea rate, the protocol was amended and the dose of abemaciclib was reduced from 200 to 150 mg. At a median follow-up of 19.5 months, median PFS was 16.4 months in the abemaciclib group as compared to 9.3 months in the standard arm (HR 0.553; 95% CI 0.449–0.681; *p* < 0.0000001).

In summary, these data again suggest that CDK4/6 inhibitors are a relevant addition to the therapeutic armamentarium in luminal breast cancer both in treatment-naïve patients as well as in patients progressing on prior endocrine therapy.

### Take home message

Based upon BrighTNess, addition of carboplatin to standard neoadjuvant chemotherapy is regarded as a meaningful option in otherwise healthy patients with TNBC. Furthermore, the combination of immunotherapy with standard chemotherapy holds promise in TNBC while the activity of single-agent immunotherapy is limited. In metastatic breast cancer patients harbouring *BRCA* germ-line mutations, olaparib therapy resulted in longer PFS and higher response rates as compared to treatment by physician’s choice. The APHINITY trial evaluating the role of dual HER2-blockade with trastzumab plus pertuzumab in the adjuvant setting indicated a small albeit significant benefit for the pertuzumab group which was mainly driven by node-positive patients while in the adjuvant treatment of hormone-receptor positive breast cancer, the SOLE trial could not establish the superiority of intermittent over standard continuous extended endocrine therapy with aromatase inhibitors. Finally, several presentations again indicated that the addition of CDK4/6 inhibitors to endocrine therapy results in a clinically relevant PFS prolongation while an OS benefit could not be established yet.
